# The Effects of Diabetes Mellitus in Patients Undergoing Off-Pump Coronary Artery Bypass Grafting

**DOI:** 10.1155/2016/4967275

**Published:** 2016-09-29

**Authors:** Yu Liu, Jinsong Han, Tao Liu, Zhonglu Yang, Hui Jiang, Huishan Wang

**Affiliations:** Department of Cardiovascular Surgery, General Hospital of Shenyang Military, Shenyang, China

## Abstract

*Objective*. To investigate the effects of diabetes mellitus (DM) in patients undergoing off-pump coronary artery bypass grafting (OPCAB).* Method*. A total of 728 patients with DM and 1380 patients without DM who underwent OPCAB treatment from March 2012 to April 2015 were reviewed. The effects of DM on intraoperative variables and postoperative complications were determined using propensity score analysis.* Results*. Two well-matched subgroups were selected using propensity score analysis (DM = 728, no-DM = 728) to compare the perioperative outcome. The duration of the ICU stay, in hours (55.2 ± 53.0 versus 49.29 ± 51.30, *P* < 0.05), postoperative new-onset atrial fibrillation (20.9% versus 14.97%, *P* < 0.05), and postoperative infection (9.2% versus 4.67%, *P* < 0.05) were greater in DM patients, as indicated by univariate analysis.* Conclusion*. OPCAB was found to be effective in DM patients, but postoperative infection and postoperative new-onset atrial fibrillation were found to be more likely to occur in DM patients than in other patients. DM was found to be a powerful risk factor for postoperative infection and postoperative new-onset atrial fibrillation.

## 1. Introduction

Coronary artery disease (CAD) and diabetes mellitus (DM) are serious diseases with substantial morbidity and mortality. DM is a well-known risk factor for the development of CAD [1], which carries the same risk of mortality as MI itself [2]. The prognosis of coronary artery disease (CAD) is significantly worse in patients with DM than in patients without DM [3].

The current best available evidence suggests that CABG may be more appropriate than percutaneous coronary interventions (PCI) for patients with CAD complicated by DM [4, 5]. Off-pump coronary artery bypass (OPCAB) surgery has been demonstrated to have a comparable, risk-adjusted mortality and to be associated with less severe complications than on-pump CABG [6, 7]. Using of this technique of OPCAB prevents the need for cardiopulmonary bypass (CPB) and avoids the serious complications that can be caused by CPB with conventional CABG, such as stroke, renal dysfunction, and systemic inflammatory response syndrome (SIRS) [8, 9]. For this reason, OPCAB is considered by many physicians to be superior to conventional on-pump CABG for patients with CAD and especially for high-risk patients [10].

The implementation of OPCAB depends on the skill and experiences of the surgeons. There have been only a few studies of OPCAB in patients with CAD complicated by DM [11, 12]. In our cardiac surgery center, most patients with CAD undergo coronary revascularization under OPCAB. This retrospective study focused on patients with and without DM undergoing OPCAB to assess the effects of DM on the postoperative complications and duration of the hospital stay.

## 2. Patients and Methods

### 2.1. Study Population

We reviewed all consecutive patients who underwent their first OPCAB in Department of Cardiovascular Surgery, General Hospital of Shenyang Military Command, from March 2012 to April 2015 in this study. This study was approved by the Ethics Committee of General Hospital of Shenyang Military Command, Shenyang city, China. All patients provided informed consent, and the study was conducted according to the ethical guidelines of the Declaration of Helsinki (1975). The study inclusion and exclusion criteria are the following.

(1) Inclusion criteria

 CAD patients with multivessel disease
 History of DM ≥ 5 years (in DM group)

(2) Exclusion criteria

 Additional cardiovascular disease requiring concomitant surgery
 Emergent surgery
 Minimally invasive operation
 Preoperative IABP for any reason
 History of DM < 5 years (in DM group)
 Infectious disease, systemic immunologic disease, malignancy, and hepatic or nephritic dysfunction

The diagnosis of DM was based on diagnostic criteria from the American Diabetes Association (ADA) [13]. Criteria for the diagnosis of diabetes are

1. A1C ≥ 6.5%: the test should be performed in a laboratory using a method that is NGSP certified and standardized to the DCCT assay,
2. FPG ≥ 126 mg/dL (7.0 mmol/L): fasting is defined as no caloric intake for at least 8 h,
3. 2 h plasma glucose ≥ 200 mg/dL (11.1 mmol/L) during an OGTT: the test should be performed as described by the World Health Organization using a glucose load containing the equivalent of 75 g anhydrous glucose dissolved in water, or
4. in a patient with classic symptoms of hyperglycemia or hyperglycemic crisis, a random plasma glucose ≥ 200 mg/dL (11.1 mmol/L).

In the absence of unequivocal hyperglycemia, criteria (1)–(3) should be confirmed by repeat testing.

### 2.2. Operative Procedure

OPCAB surgery was performed by experienced, qualified surgeons who carried out 350–400 of OPCAB cases per year. Commercially available stabilizers (Octopus IV, Medtronic Inc., MN, US) were used to provide a motionless operative field. During the OPCAB, distal anastomoses were created by proximal blocking or the use of intraluminal coronary shunts. Proximal anastomoses were performed using a partial occlusion clamp on the aorta. During the operation, circulation was kept stable by adjusting the patient's position (head tilted down or head tilted up), infusion of intravascular fluids, and use of vasoactive drugs (such as lipid nitrate, noradrenalin, and esmolol). The Portland protocol was used to keep glucose levels less than 150 mg/dL in both DM and non-DM groups [14]. All data were collected by trained clinicians.

### 2.3. Evaluation of Endpoints

The primary endpoint was mortality within a month of operation. The secondary endpoints were the occurrence of adverse events within a month of operation. These adverse events included severe arrhythmia (asystole, ventricular tachycardia, or ventricular fibrillation), heart failure, severe renal failure requiring dialysis, and any surgery or invasive procedure necessary to treat a postoperative adverse event.

### 2.4. Statistical Analysis

SPSS 17.0 software was used for the data analysis. Data are presented as a percentage or as a mean ± standard deviation. Student's *t*-test and chi-square test were performed to determine the effects of perioperative parameters on the hospital stay. To compensate for the nonrandomized design of this retrospective analysis, a propensity score analysis was performed. The analysis included a number of baseline demographic variables, such as sex, history of hypertension, and grafts needed, which had significant differences between DM and no-DM groups based on the raw data. Probability was calculated for each patient. Matching was performed using a randomization model, with the maximal difference in probability score between any paired-patients from DM and no-DM group. The propensity score was then used to select 728 well-matched pairs of patients in each group (DM = 728, no-DM = 728). *P* < 0.05 was considered statistically significant.

## 3. Results

### 3.1. Patient Demographic

Baseline demographics of both raw data and propensity score adjusted data are shown in Table 1. In raw data, a total of 728 patients with DM and 1380 patients without DM underwent OPCAB in this study. Patients with DM were more likely to be females, to have hypertension, and to need more bypass grafts. There were no other obvious differences in other preoperative characteristics or comorbidities. However, in the propensity score adjusted data, 728 patients with DM and 728 patients without DM were enrolled. In these patients, baseline demographics of DM and no-DM group were closely matched with no significant differences.

### 3.2. Intraoperative Parameters and Postoperative Complications

Intraoperative parameters and postoperative complications are shown in Table 2. Among the raw data, there was a significant increase in the duration of postoperative mechanical ventilation, duration of ICU stays, rate of postoperative infection, and rate of postoperative new-onset atrial fibrillation in the patients with DM compared to those without DM (*P* < 0.05 each). These problems lasted longer or were more intense in patients with DM. In contrast, no significant difference was found between the two groups in total volume of mediastinal tube drainage, reoperation for any reason, need for reintubation and mechanical ventilation, use of intra-aortic balloon pump (IABP) during or after the operation, renal failure, perioperative transfusion, or mortality. However, in the propensity score adjusted data, a similar trend was found to be based on the raw data, with the exception of postoperative mechanical ventilation, which showed no differences between the two groups.

### 3.3. Perioperative Blood Glucose Level

**Figure 1:**
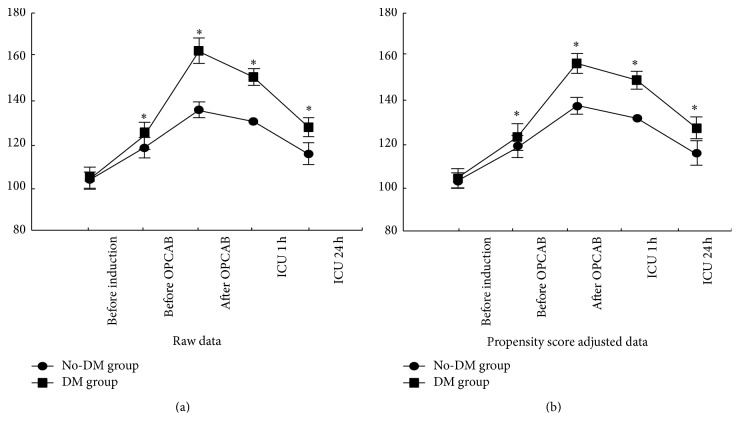
Perioperative blood glucose level. Raw data showed no difference in preoperative blood glucose concentration between the two groups caused by the excellent glucose control in the DM group. Blood glucose levels began to increase after induction and decrease after OPCAB. Blood glucose was greater in the DM group than in the non-DM group immediately before induction, before OPCAB, after OPCAB, at ICU 1 h, and at ICU 24 h. The propensity score adjusted data showed the same trends as the raw data (^*∗*^ *P* < 0.05 versus no-DM group), values are mean ± SD.

Among the raw data, there was no difference in preoperative blood glucose concentrations between the two groups (before induction), because of the excellent baseline control of blood glucose in the DM group; however, blood glucose was higher in the DM group before OPCAB, after OPCAB, and at ICU 1 h and ICU 24 h (*P* < 0.05), despite using the Portland protocol for glucose control. However, in the propensity score adjusted data, the same trend was shown as that based on the raw data (Figure 1).

## 4. Discussion

Conventional CABG with cardiopulmonary bypass is often associated with serious complications associated in part with the CPB [9]. In order to prevent potential complications caused by CPB, OPCAB has been used to treat DM patients with CAD. Emmert et al. have demonstrated that OPCAB has a lesser mortality rate and better postoperative outcomes in diabetic patients compared to conventional on-pump CABG [10]. Other reports also suggested that OPCAB was superior for high-risk patients, including patients with DM [4, 15]. In our study, the baseline and perioperative data of patients with and without DM admitted to our medical center were studied. The purpose of the present work was to determine the effects of DM on the morbidity and operative mortality of OPCAB compared to patients without DM.

The results of the current study showed that the proportion of female patients and patients with preoperative hypertension was greater than that of male patients and patients without preoperative hypertension. Baseline demographics also showed that more bypass grafts were needed in the DM group than in the non-DM group. Intraoperative and postoperative data showed that patients with DM also had longer ICU stays, a greater incidence of infection, and a greater rate of postoperative new-onset atrial fibrillation than patients without DM whether based on raw data or propensity score adjusted data. However, the postoperative mechanical ventilation showed significant differences between the two groups, as indicated by raw data rather than adjusted data. In this way, the results showed DM to be a risk factor for ICU stays, postoperative infections, and postoperative new-onset atrial fibrillation.

Patients with DM have 2-to 4-fold greater risk of developing cardiovascular disease than those without DM [16]. Moreover, DM increases the rate of atherogenesis, lipid abnormalities, and myocardial vulnerability, which can render life-threatening cardiovascular events more severe [17]. These results may explain the findings that patients with DM have a greater incidence of hypertension preoperatively, and the greater amount of coronary calcification in DM patients than in non-DM patients may explain the difference in the presence of hypertension [18]. Furthermore, these results showed that patients with DM needed more bypass grafts than those without. These findings can be explained by those of a previous study [19], which demonstrated that patients with DM often present with more diffuse coronary artery disease, including multiple vessels, than patients without DM.

In the current study, the definition of postoperative infection was inclusive of all infective complications and included wound infection in the chest incision, respiratory infection, urinary tract infection, and infections of other organs. These were confirmed by positive results of bacterial culture using blood, sputum, urine, and various secretions. The rate of postoperative infections in this study was greater than in previous studies of conventional CABG, because these studies present only sternal wound infections as postoperative infection [20, 21]. In addition, the proportion of postoperative infection in the group with DM was significantly greater than in the no-DM group (9.2% in DM group versus 4.67% in no-DM group; *P* < 0.001). DM was also shown to be a risk factor for the development of a postoperative infection, in all likelihood because of the difficulty of effective glucose control postoperatively related to a decrease in insulin secretion and an increase in insulin resistance. Markedly increased blood glucose levels have been associated with a functional decline of neutrophils. Previous studies have demonstrated that high blood glucose concentrations are an independent risk factor for mortality in patients undergoing cardiac surgery [22, 23].

New-onset atrial fibrillation is one of the most common forms of arrhythmia after heart surgery. The incidence of postoperative atrial fibrillation (POAF) in patients after coronary artery bypass grafting (CABG) surgery varies from 20% to 35% [24, 25], which can increase postoperative mortality [26]. Previous studies have demonstrated that DM is a powerful risk factor for the occurrence of POAF [27–29]. In the current study, POAF was defined as an episode of atrial fibrillation lasting more than 15 min or an episode necessitating medical treatment. Results showed that the incidence of POAF in DM group is significantly higher than in the no-DM group. Both postoperative infection and POAF were found to influence the postoperative recovery, which might be why ICU stays were longer in the DM group than in the no-DM group.

Because of these findings, perioperative glucose control has become an important consideration in postoperative care. The Portland protocol has become one of the most common protocols used to control glucose [14], and its effectiveness has been validated by other researchers [30]. In the current study, however, there was some difficulty in controlling blood glucose concentrations, likely for fear of inducing severe hypoglycemia, especially in the DM group, which might have contributed to the high rate of postoperative infection and POAF in DM group.

## 5. Limitations

The current study has some limitations. The current study was a retrospective, single-center, nonrandomized study. Although the data were adjusted with propensity score analysis, the different baseline demographics and the number of patients enrolled may have affected the quality of data and the validity of the results. A prospective study with a larger sample size and multiple centers should be performed.

## 6. Summary/Conclusion

The current study shows that OPCAB is safe in patients with DM. DM was found to be a powerful risk factor for postoperative infection and POAF. When patients with DM undergo OPCAB, care must be taken to prevent and treat postoperative infection and POAF.

## Figures and Tables

**Table 1:** Baseline demographic data between two groups (*n* [%]).

	Raw data			Propensity score adjusted data		
	DM (728)	no-DM (1380)	*P* value	DM (728)	no-DM (728)	*P* value
Age (y)	61.71 ± 9.56	62.47 ± 8.86	0.562	61.71 ± 9.56	63.04 ± 8.41	0.485
Female	223 (30.63)	317 (22.97)	0.000	223 (30.63)	223 (30.63)	1.000
Weight (kg)	71.47 ± 10.95	70.97 ± 12.66	0.361	71.47 ± 10.95	70.28 ± 12.36	0.052
BMI (kg/m^2^)	25.12 ± 3.11	25.47 ± 5.34	0.104	25.12 ± 3.11	25.10 ± 5.99	0.938
LVEF (%)	59.45 ± 5.67	59.74 ± 5.42	0.254	59.45 ± 5.67	59.75 ± 5.42	0.310
NYHA II	465 (63.87)	924 (66.96)	0.161	465 (63.87)	491 (67.44)	0.151
NYHA III	263 (36.13)	456 (33.04)		263 (36.13)	237 (32.56)	
Obsolete cerebral infarction	131 (17.99)	218 (15.80)	0.196	131 (17.99)	152 (20.88)	0.164
Obsolete pulmonary disease	204 (28.02)	367 (26.59)	0.503	204 (28.02)	227 (31.18)	0.187
History of hyperlipidemia	50 (6.87)	77 (5.58)	0.391	50 (6.87)	36 (4.95)	0.120
History of hypertension	519 (71.29)	850 (61.59)	0.000	519 (71.29)	519 (71.29)	1.000
History of smoke	362 (49.73)	798 (57.83)	0.098	362 (49.73)	388 (53.30)	0.173
Previous MI	505 (63.37)	943 (68.33)	0.657	505 (63.37)	482 (66.21)	0.197
Grafts	2.98 ± 0.133	2.96 ± 0.221	0.014	2.98 ± 0.133	2.98 ± 0.122	1.000

**Table 2:** Intraoperative and postoperative characteristics of the two groups (*n* [%]).

	Raw data			Propensity score adjusted data		
	DM (728)	no-DM (1380)	*P* value	DM (728)	no-DM (728)	*P* value
Intubation time (h)	19.72 ± 27.91	16.32 ± 33.62	0.020	19.72 ± 27.91	16.66 ± 42.08	0.103
Duration of ICU stay (h)	55.21 ± 53.02	49.83 ± 46.89	0.014	55.21 ± 53.02	49.29 ± 51.30	0.026
Total volume of drainage (mL)	762.95 ± 575.93	792.57 ± 578.14	0.264	762.95 ± 575.93	779.94 ± 564.80	0.571
Repeat operation	7 (0.96)	15 (1.09)	1.000	7 (0.96)	7 (0.96)	1.000
Repeat mechanical ventilation	12 (1.65)	14 (1.01)	0.218	12 (1.65)	8 (1.10)	0.368
IABP during and after the operation	29 (3.98)	48 (3.48)	0.544	29 (3.98)	30 (4.12)	0.894
Renal failure	3 (0.41)	4 (0.29)	0.698	3 (0.41)	3 (0.41)	1.000
Postoperative infection	67 (9.2)	64 (4.64)	0.000	67 (9.2)	34 (4.67)	0.001
Postoperative new-onset atrial fibrillation	152 (20.88)	194 (14.06)	0.000	152 (20.88)	109 (14.97)	0.003
Perioperative transfusion	208 (28.57)	381 (27.61)	0.640	208 (28.57)	221 (30.36)	0.455
Mortality	7 (0.96)	12 (0.87)	0.813	7 (0.96)	6 (0.82)	0.781
